# A privacy-preserving federated learning framework for generalizable CBCT to synthetic CT translation in head and neck

**DOI:** 10.3389/fdgth.2026.1812254

**Published:** 2026-06-15

**Authors:** Ciro Benito Raggio, Paolo Zaffino, Maria Francesca Spadea

**Affiliations:** 1 Institute of Biomedical Engineering, Karlsruhe Institute of Technology, Karlsruhe, Germany; 2 Department of Experimental and Clinical Medicine, Magna Graecia University, Catanzaro, Italy

**Keywords:** CBCT, data privacy, data sharing, deep learning, federated learning, head and neck cancer, image-to-image translation, synthetic computed tomography

## Abstract

**Background:**

Cone-beam computed tomography (CBCT) has become a widely adopted modality for image-guided radiotherapy (IGRT). However, CBCT is characterized by increased noise, limited soft-tissue contrast, and artifacts. These issues result in unreliable Hounsfield unit (HU) values, which limits electron density estimation for direct dose calculation. These issues have been addressed by deriving synthetic CT (sCT) from CBCT, particularly by adopting deep learning (DL) methods. However, existing DL approaches are hindered by institutional heterogeneity, scanner-dependent variations, and data privacy regulations that prevented multi-center data sharing.

**Methods:**

To overcome these challenges, we propose a cross-silo federated learning (FL) approach for CBCT-to-sCT synthesis in the head and neck region. This approach extends the original FedSynthCT framework to a different image modality and anatomical region. A conditional generative adversarial network (cGAN) was trained using data from three European medical centers within the SynthRAD2025 public challenge dataset while maintaining data privacy at each institution. A combination of the FedAvg and FedProx aggregation strategies, alongside a standardized preprocessing pipeline, was adopted to federate the DL model.

**Results:**

The federated model effectively generalized across participating centers, as evidenced by the mean absolute error (MAE) ranging from 64.38±13.63 to 85.90±7.10 HU, the structural similarity index (SSIM) ranging from 0.88±0.02 to 0.92±0.04, and the peak signal-to-noise ratio (PSNR) ranging from 32.86±0.94 to 34.91±1.04 dB. Notably, performance on an external validation dataset of 60 patients yielded comparable metrics: a MAE of 75.22±11.81 HU, an SSIM of 0.90±0.03 and a PSNR of 33.52±2.06, confirming robust cross-center generalization despite differences in imaging protocols and scanner types, without additional training. Furthermore, a visual analysis of the results revealed that the obtained metrics were influenced by registration errors.

**Conclusions:**

Our findings demonstrated the technical feasibility of FL for CBCT-to-sCT synthesis task while preserving data privacy, offering a collaborative solution for developing generalizable models across institutions without requiring data sharing or center-specific models.

## 1 Introduction

Radiotherapy (RT) has seen significant advancements in recent decades, particularly with the integration of image-guided radiotherapy (IGRT). IGRT involves the use of frequent imaging during treatment to account for anatomical changes and improve the accuracy of radiation delivery. Cone-beam computed tomography (CBCT) scanners are often incorporated into the gantry of linear accelerators, facilitating their integration into clinical practice. Consequently, CBCT emerged as one of the most widely adopted IGRT modalities (1–3). The advent of CBCT addressed the limitations of planar imaging, previously dominant in early image guidance, by enabling volumetric imaging directly within the treatment suite, significantly improving treatment precision and clinical workflows (2, 4, 5). This technological evolution enabled improved alignment, visualization of patient anatomy, and real-time verification of target localization, allowing clinicians to manage both inter- and intra-fractional anatomical variability. As a result, CBCT became a pivotal technology in the development of online image-guided adaptive radiotherapy (IGART) approaches (4–7). Despite these advantages, CBCT is hindered by several drawbacks, including increased image noise, reduced soft-tissue contrast, and reconstruction artifacts, which lead to unreliable Hounsfield Unit (HU) values and limit its applicability in electron density estimation for direct dose calculation (1, 5, 6). To overcome these limitations, the concept of synthetic CT (sCT) generation was introduced, where CBCT images are translated into equivalent synthetic CT images with improved dosimetric fidelity. This approach avoids unnecessary radiation exposure and reduces clinical workload by eliminating the need for additional scans (3, 5, 8).

The CBCT-to-sCT conversion task has evolved considerably, with applications across diverse anatomical regions, including head and neck, thorax, pelvis, prostate, abdomen, and pancreas, highlighting both its broad clinical relevance and intrinsic methodological challenges (3, 9). Among the strategies developed for sCT generation, deep learning (DL) approaches have proven especially effective, demonstrating strong capabilities in learning complex, non-linear mappings between image domains and producing high-quality synthetic images suitable for adaptive RT applications (1, 3, 5, 8).

Convolutional Neural Networks (CNNs) have been widely adopted in medical image analysis due to their ability to extract both local and global features, making them well suited for enhancing CBCT image quality in CBCT-to-sCT synthesis tasks. In this context, the U-Net architecture (10), has demonstrated notable efficacy in image-to-image translation tasks by enabling pixel-level predictions while preserving spatial and contextual information (3, 9). Generative Adversarial Networks (GANs) have also been extensively employed, particularly conditional GANs such as Pix2Pix (11), which utilize a generator-discriminator framework and a PatchGAN discriminator to promote local structural consistency and realistic texture synthesis. Cycle-GAN architectures further introduced cycle consistency constraints to preserve anatomical structures during CBCT-CT translation, although training instability has been reported (3, 9). More recent approaches have explored Transformer-based architectures and Denoising Diffusion Probabilistic Models (DDPMs) for sCT generation. Transformer-based models, such as TransCBCT (12), employ self-attention mechanisms to capture long-range spatial dependencies and complex anatomical relationships, but require large training datasets, substantial computational resources, and are sensitive to image resolution (3, 12). DDPMs, which learn to reverse a progressive noising process, have demonstrated high-fidelity sCT generation when conditioned on CBCT inputs, but are similarly computationally intensive and characterized by slow inference and high hardware requirements (3). Despite their architectural differences, all of the aforementioned models have demonstrated promising performance in producing clinically viable sCTs for radiotherapy planning (3, 9).

The evaluation of sCT generation DL models in predicting accurate images comparable to gold standard planning CTs, also named ground-truth CT, was assessed by computing metrics such as mean absolute error (MAE), mean squared error (MSE), peak signal-to-noise ratio (PSNR), and structural similarity index (SSIM), computed within patient-specific masks to exclude background regions. Among these metrics, the MAE was often used as loss function, and was referred to as masked MAE or L1 Loss (3, 9). Gradient descent-based optimization has been extensively adopted for the iterative update of model weights. Concurrently, pre-processing methodologies such as image registration, image normalization and data augmentation have been demonstrated to be critical for model performance and stability (3, 8, 9, 13).

Despite the increasing adoption of DL for the sCT generation, its application remains limited by institutional heterogeneity, scanner-dependent variations, and heterogeneous imaging protocols, often necessitating single-site model training and constraining clinical scalability (3, 7, 14). Although multi-center data aggregation could partially address these limitations, it is severely constrained by strict data privacy regulations, such as the General Data Protection Regulation (GDPR)^1^ in Europe, and the Health Insurance Portability and Accountability Act (HIPAA)^2^ in the United States, which restrict the sharing and centralization of sensitive medical data (14–16). These limitations emphasize the necessity for collaborative solutions that facilitate access to diverse and extensive datasets without necessitating direct data sharing or centralized storage.

In this context, federated learning (FL) has emerged as a decentralized training paradigm that enables institutions to collaboratively train DL models while keeping data local, thereby maintaining compliance with privacy regulations and enhancing cross-center collaboration (17–19). FL has gained increasing attention in medical imaging, with applications in classification and segmentation tasks (16, 17, 20–23). Beyond the domains of classification and segmentation, federated approaches recently emerged in the context of medical image synthesis. For instance, FedMed-GAN has been developed to synthesize cross-modality brain magnetic resonance images while leveraging simple federated aggregation techniques such as the federated averaging (FedAvg), which averaging the locally updated model weights from multiple clients to obtain a global model without sharing raw data (24, 25).

FedSynthCT-Brain was the first study to demonstrate the successful application of FL to the sCT synthesis task, specifically from brain T1-weighted magnetic resonance imaging (MRI) data, without compromising performance on external datasets (14). This finding suggested potential applications for extending the federated concept to the context of CBCT-to-sCT translation, where the development of robust, center-agnostic and generalizable models is essential for clinical adoption (8).

More in details, while MRI-to-CT translation involves inferring HU values from images which cannot inherently encode them, CBCT measures x-ray attenuation but suffers from severe quality degradation. Thus, the main challenge in the CBCT-to-CT translation task shifts from learning a cross-modality mapping to restoring accurate HU values from noisy, scattered and vendor-dependent reconstructed images. Furthermore, the transition of the model working region, from the brain (introduced by FedSynthCT-Brain) to the head and neck, introduces greater anatomical variability and high-contrast structures, such as dental implants and extensive air cavities. Consequently, this transition results in a task that is substantially different and more sensitive to misalignment and residual registration errors. These factors make the successful deployment of FL in this setting non-straightforward and highlight the need for a dedicated framework to determine whether federated training can remain robust and viable under such adverse and heterogeneous conditions.

Therefore, in this study, we proposed a cross-silo horizontal FL approach for CBCT-to-sCT in the head and neck region, extending the FedSynthCT (14) framework to a different imaging modality and a larger anatomical region. A federated DL model was collaboratively trained using data from different European centers of the public SynthRAD2025 challenge dataset (26). The approach was validated not only on each federated center but also on an independent dataset outside the federation. This validation process was used to assess model generalization capabilities, thereby demonstrating that the proposed approach can result in more robust and generalizable models while preserving data privacy.

## 2 Materials and methods

### 2.1 Datasets

The datasets employed in this work were from the publicly available SynthRAD2025 challenge repository (26, 27). The institutions (also referred to as centers or clients) focus on head and neck (HN) region and were labeled as centers A, B, C, and E. Center D was deliberately excluded from this study due to the limited availability. For consistency, we adopted the same naming convention for the centers as used in the challenge.

As reported, a few thorax scans were included in the HN cohort (26); these outlier cases were excluded from the study. The final number of subjects included per center is reported in Table 1. For each subject, the dataset included a CBCT scan, a CT scan, and a binary segmentation mask delineating the patient body.

**Table 1:** Overview of the key CBCT and CT original acquisition parameters used at Centers A, B, C, and E according to the dataset documentation (26).

Parameter	Center A	Center B	Center C	Center E
CBCT				
Patient #	60	65	63	63
Scanner	Elekta XVI v5.x	Elekta XVI v5.52	Elekta XVI v5.x	Varian TrueBeam OBI
kVp	100–120	100	120	100–125
Exposure Time [ms]	10–32	10	22	7,500–18,060
Rows/Columns	270	270	270–512 × 270–512	512 × 512
Pixel spacing [mm]	1 × 1	1 × 1	1 × 1	0.5–0.9 × 0.5–0.9
Slice thickness [mm]	1	1	1	2
CT				
Patient #	60	65	63	63
Scanner	Philips Big Bore (90), Siemens Biograph40 (10)	Toshiba Aquilion/LB	Philips Brilliance Big Bore (93), Siemens Biograph40 (7)	Toshiba Aquilion/LB, Siemens Biograph128
kV	120	120	120	120
Exposure Time [ms]	615–1,000	500–1,000	922–1457	500–1,000
Rows/Columns	512	512	512	512
Pixel spacing [mm]	0.7–1.4 × 0.7–1.4	1.1–1.4 × 1.1–1.4	1–1.2 × 1–1.2	1–1.5 × 1–1.5
Slice thickness [mm]	2–3	1–3	2–3	3–5

Variability in imaging protocols and scanner types among relevant centers has been identified. Notably, CBCT scans from Center E were acquired using a different system, in contrast to the devices employed in centers A, B and C. Center E also exhibited markedly longer exposure times and higher in-plane resolution variability. Regarding the CT images, it was observed that all centers employed standard 120 kVp acquisitions; however, exposure time, slice thickness and pixel spacing varied.

### 2.2 Federation setup and pre-processing

**Figure 1:**
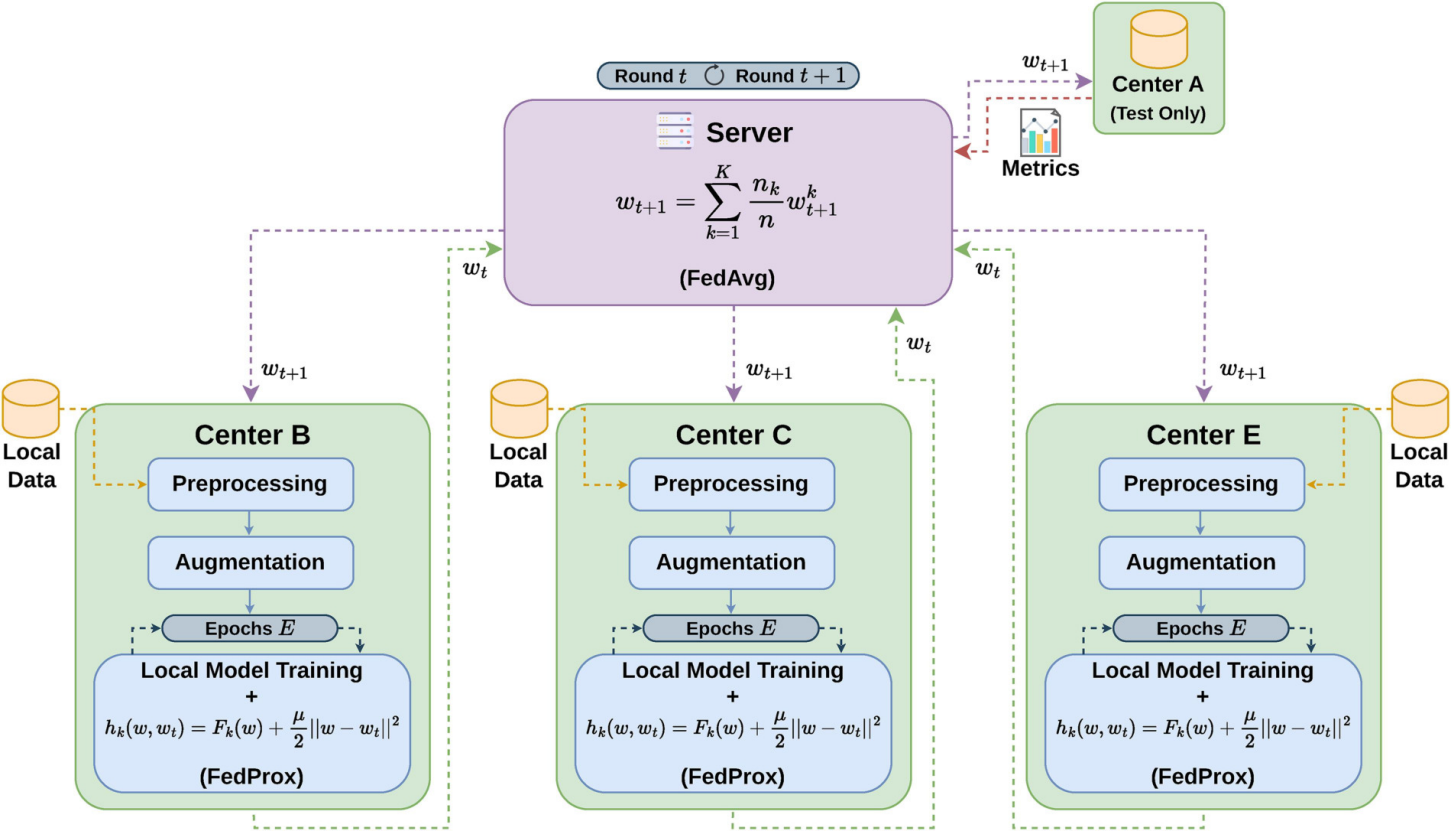
Schematic overview of the proposed FL framework. Three clients (Centers B, C, and E) implement a standardized preprocessing and data augmentation pipeline for their local datasets. Each client k performs local model training at round t, introducing the FedProx regularization. The locally updated weights ($w_{t}$) are subsequently transmitted to a central server, where they are aggregated via FedAvg. The resulting global model ($w_{t+1}$) is subsequently redistributed to all clients for the next round. The Center A dataset is exclusively reserved for external validation, thereby enabling an evaluation of the federated model’s generalization performance.

The federated training setup included a central server associated with Center A dataset and three clients from Centers B, C, and E. An overview of the proposed FL framework is illustrated in Figure 1. The federated infrastructure was implemented using Flower (28), PyTorch (29) and MONAI (30). The federated training followed a cross-silo, synchronous, and full-participation scheme. At each global round, all clients performed local updates simultaneously, and the server aggregated the global model parameters only after receiving updates from every client. Experiments were conducted on a single-node machine with an NVIDIA A100 (80GB), 16 CPU cores, and 128GB RAM. While all experiments were conducted within a single-node environment for prototyping purposes, the FL framework incorporated all the necessary components to replicate a real-world federated deployment, including encrypted communication using TLS certificates, client reconnection strategies, and the ability to resume training from saved checkpoints.

The Center A dataset was reserved exclusively for external validation, enabling an evaluation of the federated model’s generalization performance. Within each participating client, four validation patients and four test patients were held out, while the remaining cases were used to train local models. This allocation reflects a trade-off between maximizing client training data and maintaining a diagnostic set for monitoring local fitting behavior, with the primary generalization assessment assigned to the external cohort from Center A. Overall, of the 251 patients included in the study, 167 contributed to federated training (≈66.5% of the total data), 24 were allocated to local validation and test sets across the three clients (≈9.5% of the total data), and 60 served as the independent external test cohort, representing ≈24% of the total data.

Since data were released for a public challenge, several pre-processing operations were applied by the authors prior to the publication, such as isotropic resampling, defacing, and rigid registration between modalities, resulting in residual anatomical misalignments between CBCTs and CTs (26). To mitigate the impact of these residual misalignments on model evaluation, an additional deformable registration step was applied exclusively to the test patients.

To ensure consistent spatial resolution across the federation, each client applied a standardized pre-processing pipeline to locally adapt data to a 256×256×256 image grid dimension. This decentralized approach –which avoided any inter-client data exchange– involved conditional cropping followed by resizing and constant-padding as needed. Furthermore, an intensity processing was applied. A local normalization process was performed on each client for each CBCT scan, employing a LUT-based normalization method proposed by Vicario et al. (31) with a fixed intensity range of [−800, 2,000]. Subsequently, CT scans were clipped to the range [−1,000, 1,500] to mitigate the impact of extreme values introduced by metal implants. These ranges were empirically chosen by analyzing the CBCTs and CTs intensity distributions available at each client site, without exchanging any images.

This preliminary harmonization step allowed for the approximation of a common intensity range across clients while preserving the constraints of the federated paradigm.

### 2.3 Deep learning model

The DL architecture employed relied on the cGAN paradigm, and more specifically, on the original Pix2Pix (11) architecture.^3^ Pix2Pix has been identified as one of the most widely adopted architectures for the CBCT-to-sCT translation task, as indicated in Section 1. The adversarial paradigm exhibited a remarkable capacity in managing imperfectly paired or partially aligned data, a prevalent scenario in clinical practice where precise voxel-wise correspondence between images cannot be assured, even after the registration process. This has led to the identification of Pix2Pix as a suitable and practical solution for our application setting.

The generator was structured as a 2D U-Net with eight encoder-decoder blocks connected via skip connections, and included dropout layers to mitigate overfitting. The discriminator was implemented as a 2D PatchGAN, composed of convolutional blocks combining 2D convolutions, instance normalization, and LeakyReLU activations to assess local realism at the patch level.

The training of the DL model was implemented using the Random-Multi2D sampling approach, to enhance the robustness and generalisability. This approach consisted of presenting to the model different axial, sagittal, and coronal slices in a randomized order, ensuring that the DL model received non-sequential input from multiple anatomical planes (14).

In detail, the discriminator’s objective (Equation 1) was achieved by minimizing the sum of two sigmoid cross-entropy losses:

$$L_{\text{D}}=\frac{1}{2}\times [\mathrm{BCE}(D(CT,CBCT),1)+\mathrm{BCE}(D(sCT,CBCT),0)]$$ (1)

where BCE(⋅) was the cross-entropy loss output, D(⋅) was the discriminator output, and 1 and 0 were images of ones and zeros indicating real and fake labels, respectively. The first term penalized misclassifications of CTs as fake, while the second term penalized misclassifications of sCTs as real.

The adversarial loss function employed for the optimization of the generator (Equation 2) was combined by two components. The first was a sigmoid cross-entropy loss that enabled the discriminator to classify the generated images as real or fake:

$$L_{\text{GAN}}=\mathrm{BCE}(D(sCT,CBCT),1).$$ (2)

The second term was the L1 loss (Equation 3) , specifically the Masked MAE (see Equation 7)—typically employed for this task as reported in Section 1—between the generated sCT and the ground-truth CT, which enforced structural similarity and penalized large deviations. This loss was also referred to as pixel loss:

$$L_{\text{\textbackslash{},pixel}}=\mathrm{MAE}(CT-sCT).$$ (3)

The total generator loss (Equation 4) was subsequently computed as:

$$L_{\text{G}}=L_{\text{GAN}}+\lambda_{\text{\textbackslash{},pixel}}\cdot L_{\text{\textbackslash{},pixel}},$$ (4)

where $\lambda_{\text{\textbackslash{},pixel}}=100$ as reported in the original Pix2Pix study (11).

Inference was based on the median voxel-voting mechanism. Specifically, following the generation of the slice-wise predictions along each anatomical plane, the final sCT volume was computed by taking the median across the three orientations (1, 14). Both training and inference strategies were previously validated in the federated scenario within the FedSynthCT-Brain framework (14).

In each local client, the model was trained using a batch size of 32 slices before participating in a federated aggregation round. The Adam optimizer with a learning rate of $10^{-4}$ was used. An additional investigation was conducted to establish the optimal number of local epochs, comparing 1, 2 and 3 epochs per round.

To further improve robustness and cross-site generalization, a consistent data augmentation pipeline was applied at the client level. Each client independently introduced random geometric and intensity transforms during the training phase, including flipping, rotations, translations, and random rescaling or shifting of intensity values.

### 2.4 Aggregation strategy and sensitivity analysis

The combination of FedAvg and FedProx (32) was employed as aggregation strategy for this study. The aggregation was equivalent with the approach used in the FedSynthCT-Brain study, as it was identified as the optimal aggregation strategy, yielding results that are equivalent to results obtained by alternative strategies while requiring a reduced number of rounds (14). Nevertheless, in order to determine the optimal proximal coefficient μ and to verify that the hyperparameter choice originally established in the FedSynthCT-Brain study (14) remains appropriate for the CBCT-to-sCT synthesis task, a sensitivity analysis was conducted.

The FedAvg algorithm was implemented on the server side to aggregate the clients’ weights, ensuring that the contribution of each client was proportional to the size of its dataset. Therefore, in the Equation 5, K represents the total amount of clients; $n_{k}$ denotes the number of samples for a single client k; n is the total amount of samples across the federation, and $w_{t+1}^{k}$ represents the post-training weights of the client k.

$$w_{t+1}=\sum_{k=1}^{K}\frac{n_{k}}{n}w_{t+1}^{k}$$ (5)

The FedProx method was subsequently implemented on the client side, thereby introducing a proximal term, denoted by $\frac{\mu }{2}\|w-w_{t}{\|}^{2}$ to the local objective function (thus, the Masked MAE loss). This term penalized large deviations from the global model weights in order to control local update variations. The Equation 6 provides a formal explanation of the impact of the proximal term on the client model. The original local objective is denoted by $F_{k}(w)$, the pre-training global model weight by $w_{t}$, the weights during local training by w, and the proximal coefficient by μ. Consequently, FedProx mitigates update divergence and approximates FedAvg when μ=0, as evidenced in the original FedProx study (32).

$$h_{k}(w,w_{t})=F_{k}(w)+\frac{\mu }{2}\|w-w_{t}{\|}^{2}$$ (6)

To assess the sensitivity of the aggregation strategy to the proximal coefficient μ, four configurations were evaluated: (i) μ=0 (FedAvg), (ii) μ=1, (iii) μ=3, and (iv) μ=5. All other hyperparameters (presented in Section 2.3) were held constant across configurations. Each experiment was run for a total of 50 global rounds, ensuring sufficient training time for convergence and enabling fair comparisons across different μ configurations.

### 2.5 Evaluation

The performance of the federated model was evaluated using standard image similarity metrics—presented in Section 1—on the external test dataset (Center A) and the client test datasets (Centers B, C, and E). Specifically, the following metrics were employed:

- **Masked Mean Absolute Error**:
  $$\text{MAE}=\frac{1}{n}\sum_{i=1}^{n}\left|CT_{i}-sCT_{i}\right|,$$ (7)
  where n is the number of voxels within the region of interest (ROI).
- **Masked Structural Similarity Index Measure (SSIM)**:
  $$\text{SSIM}=\frac{\left(2\mu_{\text{sCT}}\mu_{\text{CT}}+C_{1})(2\sigma_{\text{sCT, CT}}+C_{2}\right)}{\left(\mu_{\text{sCT}}^{2}+\mu_{\text{CT}}^{2}+C_{1})(\sigma_{\text{sCT}}^{2}+\sigma_{\text{CT}}^{2}+C_{2}\right)},$$ (8)
  where the mean (μ), variance/covariance (σ), and constants $C_{1}=(k_{1}L{)}^{2}$, $C_{2}=(k_{2}L{)}^{2}$ are computed over the ROI. Here, L is the dynamic range of the CT image, with $k_{1}=0.01$ and $k_{2}=0.03$.
- **Masked Peak Signal-to-Noise Ratio (PSNR)**:
  $$\text{PSNR}=10\cdot \log_{10}\left(\frac{MAX_{CT}^{2}}{\text{MSE}}\right),$$ (9)
  where $MAX_{CT}$ is the maximum intensity value in the CT image and MSE is the mean squared error between CT and sCT within the ROI.

In order to assess the impact of registration errors on image similarity metrics, a registration quality analysis was conducted on the external test cohort from Center A, which was sufficiently large to enable a correlation analysis. For each patient, the Normalized Mutual Information (33) (NMI) between the CT and CBCT volumes was computed both before and after the additional deformable registration step. Thus, the NMI was used an intensity-based surrogate for registration quality. The patient-specific variation in NMI was defined as:

$$\Delta \text{NMI}={\text{NMI}}_{\text{after}}-{\text{NMI}}_{\text{before}}$$ (10)

This variation was then correlated with the corresponding change in generation error:

$$\Delta \text{MAE}={\text{MAE}}_{\text{after}}-{\text{MAE}}_{\text{before}}$$ (11)

where ${\text{MAE}}_{\text{after}}$ and ${\text{MAE}}_{\text{before}}$ denote the MAE (see Equation 7), obtained by using as input the CBCT volumes after and before the deformable registration step, respectively.

## 3 Results

The performance of the proposed federated cGAN model across multiple clinical centers was assessed through both quantitative and qualitative analyses, as reported in Table 2 and Figures 3–6, respectively.

**Table 2:** Quantitative performance across all centers for the four proximal coefficient μ configurations evaluated in the sensitivity analysis.

Center	Configuration	μ	MAE [HU]	SSIM	PSNR [dB]
Center A (external)	FedAvg	0	75.17±12.54	0.90±0.04	33.63±2.18
	FedAvg + FedProx	1	75.57±12.61	0.90±0.04	33.61±2.14
	FedAvg + FedProx	3	75.22±11.81	0.90±0.03	33.52±2.06
	FedAvg + FedProx	5	75.80±12.51	0.90±0.04	33.59±2.13
Center B	FedAvg	0	81.92±5.89	0.88±0.02	33.09±0.93
	FedAvg + FedProx	1	82.22±6.11	0.88±0.02	33.07±0.93
	FedAvg + FedProx	3	85.90±7.10	0.88±0.02	32.86±0.94
	FedAvg + FedProx	5	82.15±6.30	0.89±0.02	33.06±0.88
Center C	FedAvg	0	64.75±14.38	0.92±0.04	34.38±1.38
	FedAvg + FedProx	1	65.02±14.43	0.92±0.04	34.38±1.35
	FedAvg + FedProx	3	64.38±13.63	0.92±0.04	34.48±1.39
	FedAvg + FedProx	5	65.86±14.68	0.92±0.04	34.27±1.37
Center E	FedAvg	0	75.55±13.40	0.91±0.02	34.74±1.03
	FedAvg + FedProx	1	74.71±12.93	0.92±0.01	34.81±0.98
	FedAvg + FedProx	3	74.22±13.23	0.92±0.01	34.91±1.04
	FedAvg + FedProx	5	74.14±12.49	0.92±0.01	34.90±0.98

Results are reported as mean ± standard deviation. Center A was used exclusively for external testing, while Centers B, C, and E participated in the federated training.

**Figure 2:**
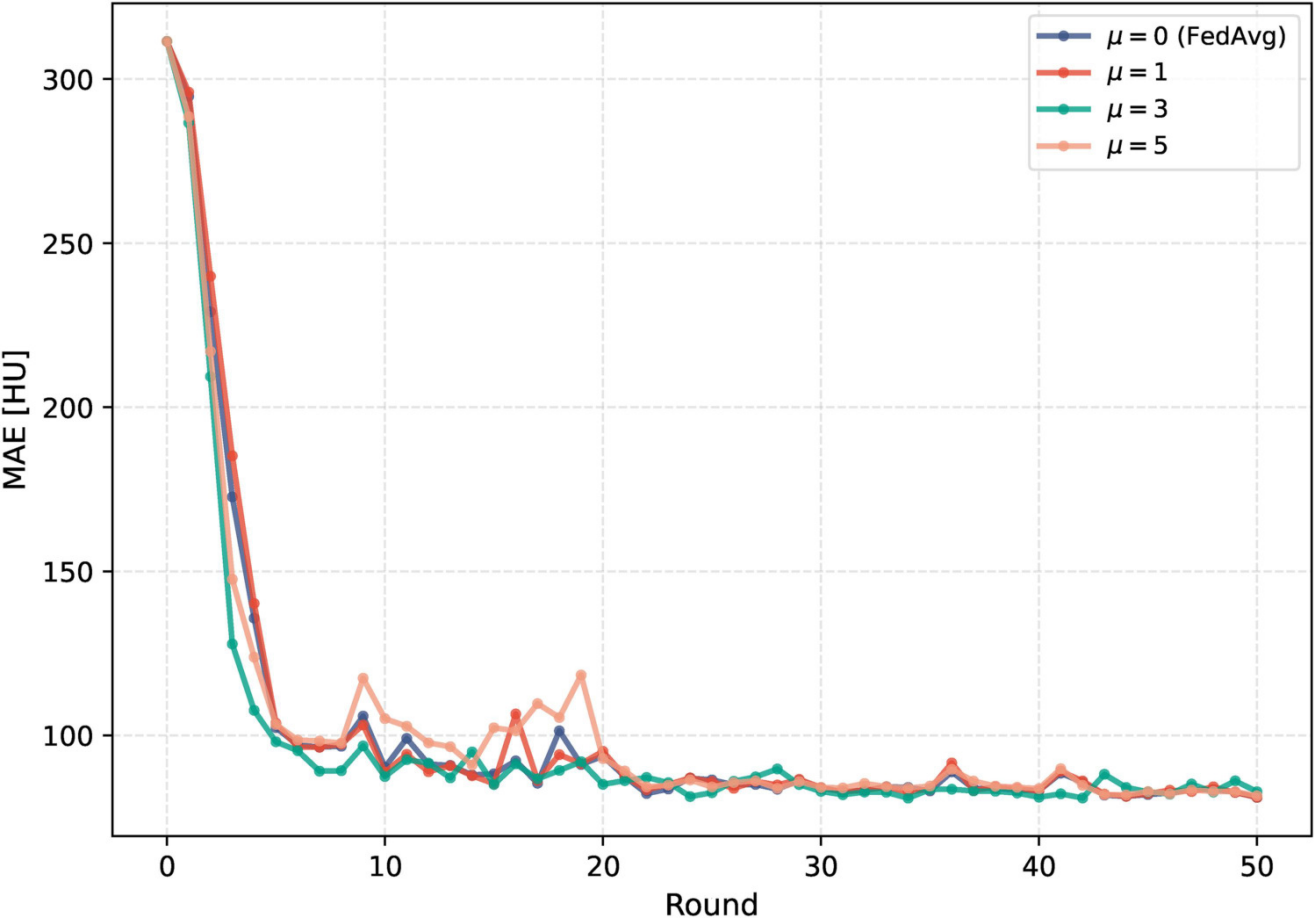
Sensitivity analysis of the proximal coefficient μ. All configurations exhibit a rapid convergence within the first 5–10 rounds, followed by a stable plateau after approximately round 20–30. The configuration of μ=3 yields the most stable and rapid convergence trend, with reduced oscillations compared to μ=5, and a faster initial descent relative to μ=0 and μ=1.

The convergence curves obtained across different μ configurations (μ=0,1,3,5) are presented in Figure 2. In all configurations, the global MAE demonstrated a rapid decrease during the initial 5–10 global rounds, from ≈310 HU to values in the range of 90–130 HU, followed by a stable plateau beyond round 30–35, with round-to-round fluctuations remaining below ±5 HU. The quantitative metrics across all configurations demonstrated high consistency (Table 2), with marginal variations in MAE, SSIM (Equation 8) and PSNR (Equation 9). Among the tested configurations, μ=3 exhibited the most stable convergence trajectory, with reduced oscillations during the intermediate rounds compared to μ=5, and a faster initial descent relative to μ=0 (FedAvg) and μ=1. Therefore, the μ=3 configuration was selected on the basis of its convergence properties, rather than on marginal differences in image quality metrics.

Subsequently, an additional investigation on the optimal number of local epochs was conducted (see Section 2.3). The convergence curves and quantitative metrics are provided in Supplementary Figure S1 and Supplementary Table S1, respectively. The federated training with a single local epoch led to a notable decrease in the stability of the global model convergence, exhibiting increased oscillations across rounds. As the number of local epochs per round increased (2 and 3), a proportional increase in computation time was observed, along with a progressively faster early descent and smaller oscillations in the intermediate rounds. Furthermore, a comparable outcome was observed in configurations with 2 and 3 local epochs, where convergence plateau was reached around round 30. However, no substantial differences were observed in the image similarity metrics. Indeed, MAE differences across centers ranged between 1–4 HU, with no single configuration consistently outperforming the others across all centers (see Supplementary Table S1). Consequently, given that increasing the number of local epochs failed to yield a substantial improvement in performance and instead led to an increase in the computational load on local clients, while a single epoch introduced instability in global convergence, adopting two local epochs was determined to be an optimal trade-off, balancing the stability of the global convergence trend, the performance of the federated model, and the computational cost on clients.

Therefore, all the subsequent per-center results reported herein refer to the μ=3 and 2 local epochs configuration.

**Figure 3:**
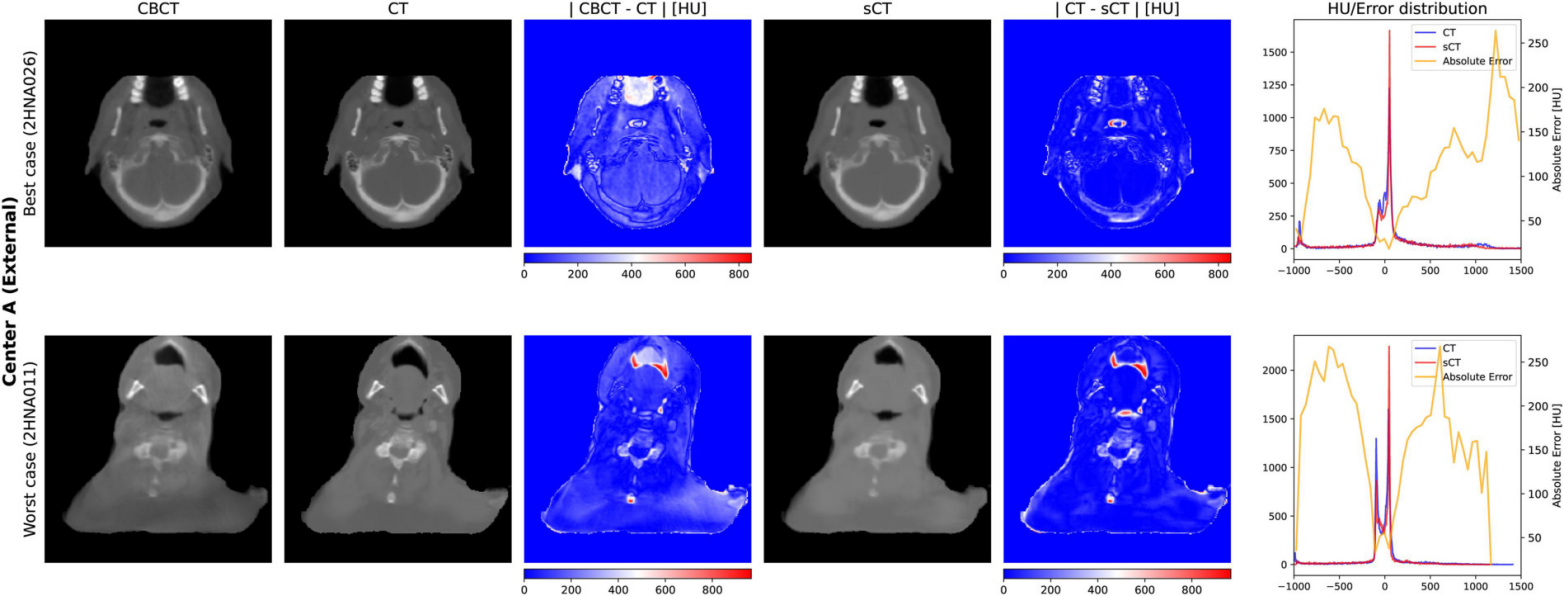
Visual evaluation of the best and worst sCT cases from Center A, used exclusively as an external test set. For each case, the mid-axial slice of the original CBCT, the ground-truth CT, the generated sCT, and the absolute difference maps for CBCT-to-CT and sCT-to-CT (error map) are shown. Additionally, intensity distributions of the sCT and CT for the displayed slice are presented, overlaid with the pixel-wise MAE. These distributions highlight the conversion accuracy across different tissue density ranges. Notably, regions with very high absolute differences appear in both CBCT-to-CT and error maps, suggesting the presence of residual misregistration between CBCT and CT scans.

As detailed in Section 2.2, Center A dataset was excluded from the federated training process and served as an external test set, including 60 test cases that provided a more robust and less biased estimation of the model’s generalization capabilities. Centers B, C, and E contributed data to train the federated model, with each center reserving four patients for local evaluation. As shown in Table 2 (for μ=3), despite not participating in the federated training, Center A achieved performance metrics comparable to those of the training centers, with a MAE of 75.22±11.81 HU, an SSIM of 0.90±0.03, and a PSNR of 33.52±2.06 dB. A qualitative evaluation of the best and worst test cases from Center A (Figure 3) showed that the generated sCTs aligned closely with the ground-truth CTs. The error maps, computed as |CT−sCT|, indicated that most deviations were within ±100 HU. The absolute difference between the original CBCT and the ground-truth CT, reported as |CBCT−CT| in Figure 3, highlighted spatial discrepancies mainly related to anatomical variations and registration mismatches.

**Figure 4:**
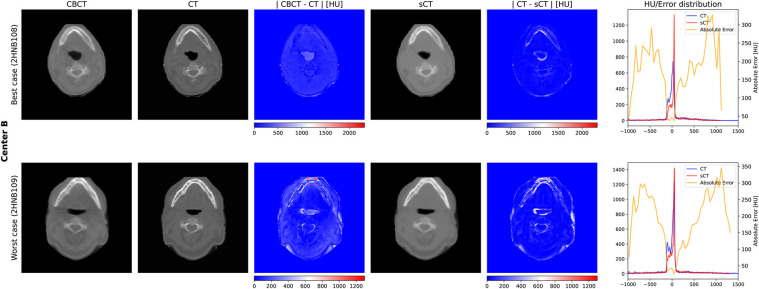
Qualitative results for the best and worst sCT cases from Center B, which showed lower image similarity metrics among the training centers. Widespread registration artifacts between CBCT and CT scans contributed to the visible errors. This was especially evident in the oral cavity of the 2HNB109 patient, and are reflected in both |CBCT−CT| figure and error map.

Among the federated clients, Center B obtained a MAE=85.90±7.10 HU, an SSIM=0.88±0.02, and a PSNR=32.86±0.94 dB. Qualitative results (Figure 4) demonstrated that the generated sCTs were anatomically consistent with the ground-truth CTs, particularly in the representation of major structures.

**Figure 5:**
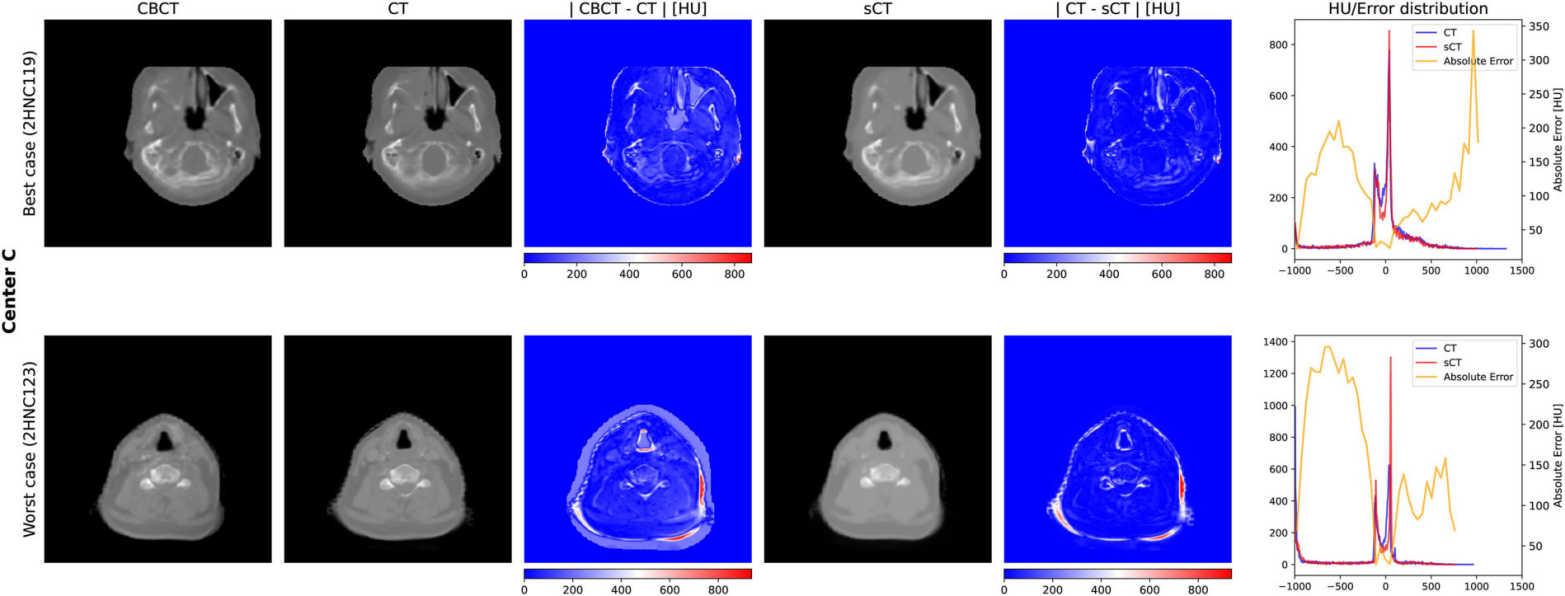
Visual comparison of the best and worst sCT cases from Center C. Compared to the other centers, Center C showed fewer registration-related errors and better image similarity, as evidenced by both the error maps (|CT−sCT|) and the distribution plots. However, case 2HNC123, identified as the worst-performing example, exhibited evident misalignments prior to the sCT generation despite the additional registration process.

Center C yielded the best overall quantitative performance, with the lowest MAE (64.38±13.63 HU), highest SSIM (0.92±0.04), and highest PSNR (34.48±1.39 dB). The worst-case example (2HNC123) shown in Figure 5 showed that the dominant discrepancies were associated with misalignments between CBCT and CT rather than pure generation errors.

**Figure 6:**
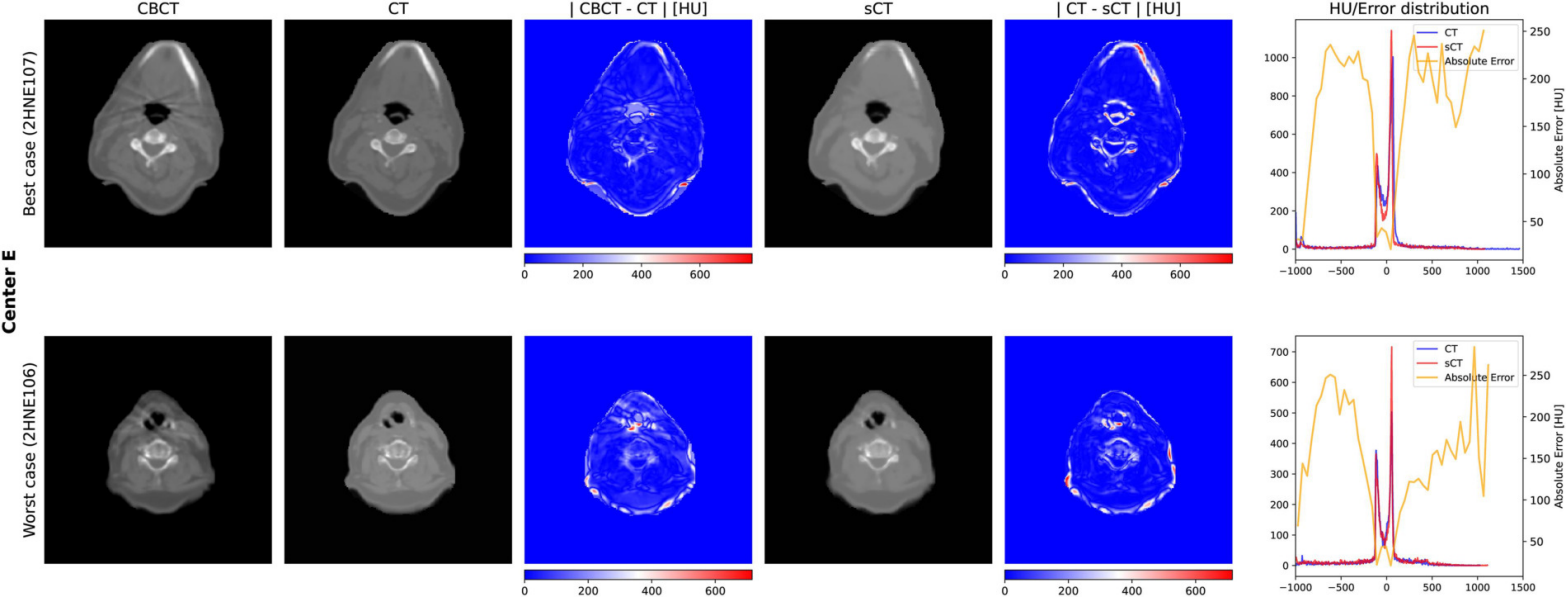
Visual evaluation of the best and worst sCT cases from Center E. Despite the use of a different scanner and acquisition protocol, the model maintained strong performance, with distribution plots indicating consistent HU alignment and localized discrepancies, mostly in air and bone regions due to inter-scan misalignments.

Center E achieved results consistent with the overall federation despite images being acquired using a different scanner and acquisition protocol (Table 1). The federated model achieved a MAE=74.22±13.23 HU, an SSIM=0.92±0.01, and a PSNR=34.91±1.04 dB (Table 2). Furthermore, quantitative results confirmed the quality of the generated sCTs, as shown in Figure 6.

**Figure 7:**
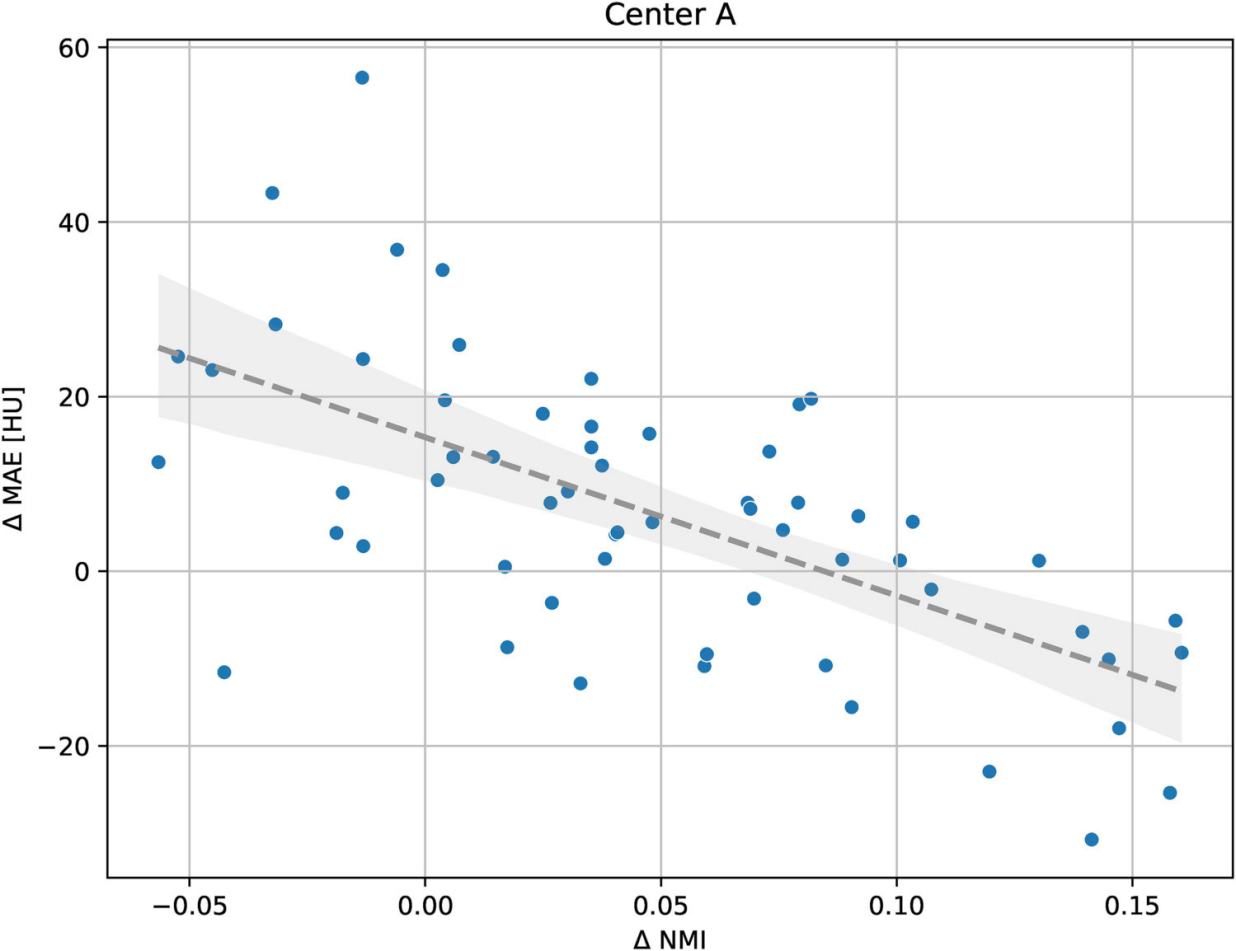
Correlation between ΔNMI and ΔMAE (HU) for the Center A test cohort before and after the deformable image registration. A significant negative correlation was observed (Pearson r=−0.63, $5.95\times 10^{-8}$; Spearman ρ=−0.61, $p=2.35\times 10^{-7}$). This finding indicates that enhancements in deformable registration, consequently leading to enhanced intensity correspondence between CT and CBCT, are associated with a decrease in MAE.The deterioration of NMI corresponds to MAE increases of ≈20−60 HU (upper-left area). In contrast, cases for which NMI improved substantially ΔNMI≈10%−15% exhibit a consistent MAE reduction (lower-right area).

The results of the registration quality analysis described in Section 2.5 are presented in Figure 7. A significant negative correlation between ΔNMI (Equation 10) and ΔMAE (Equation 11) was observed in the Center A cohort, with a Pearson coefficient of r=−0.63 ($p=5.95\times 10^{-8}$) and a Spearman coefficient of ρ=−0.61 ($2.35\times 10^{-7}$).

## 4 Discussion

In this work, we investigated whether a cross-silo FL framework can support robust CBCT-to-sCT synthesis in the head and neck region under realistic multi-center constraints. Specifically, we extended the FedSynthCT (14) study to a different imaging modality and a more anatomically heterogeneous region, evaluating its performance across three federated European centers and an independent external validation cohort from the SynthRAD2025 dataset (27). By analyzing both quantitative metrics and qualitative image characteristics, we aimed to determine whether federated training can preserve generalization capability despite inter-institutional variability in scanners, acquisition protocols, and anatomical presentation, without requiring data sharing or center-specific retraining.

A sensitivity analysis was conducted on the proximal coefficient μ, which confirmed the suitability of μ=3 for the current task in accordance with the findings of FedSynthCT-Brain (14), despite the acknowledged differences in imaging modality, anatomical region, and scanner heterogeneity. As demonstrated in Figure 2, the convergence behavior exhibited that moderate regularization (μ=3) accelerated and stabilized convergence in comparison to FedAvg (μ=0), μ=1 and μ=5. Indeed, for μ<3 the convergence was slower and exhibited greater fluctuations in the intermediate rounds (approximately rounds 9–20), while more severe regularization (μ=5) introduced instability in the intermediate rounds, delaying optimization. Consequently, this finding indicates that proximal regularization is beneficial, but leads to over-constraining local updates and hinders global convergence when severe. Notably, the minimal variations in the final image quality metrics across all μ configurations (Table 2), suggest that the convergence benefit of μ=3 occurs without compromising final performance. Furthermore, the consistent plateau behavior observed across all configurations demonstrated that 50 rounds were sufficient to ensure stable convergence of the federated model.

The local epochs analysis revealed that a single local epoch introduced instability and slowed convergence (see Supplementary Figure S1). The configurations with 2 and 3 local epochs yielded fewer oscillations across rounds. However, no substantial improvement in image quality metrics was observed, with MAE differences ranging from 1 to 4 HU across centers and no consistent hierarchy among configurations. Moreover, minimizing local updates in the context of FL is typically advantageous to mitigate client drift (24). Although FedProx partially constrains local divergence, its regularizing effect could diminish as the number of local epochs increases (32). Consequently, 2 local epochs were selected as the configuration that optimally balanced convergence stability, model performance, and computational cost on local clients.

External validation on Center A enabled further investigation into the generalization capability of the federated model in a fully independent setting. The observed performance metrics were comparable to those obtained across the participating centers, despite the absence of additional training or fine-tuning. These findings suggest stable model behavior on previously unseen data. Furthermore, qualitative analysis indicated that residual spatial discrepancies (Figure 3) between CBCT and planning CT were predominantly associated with registration inaccuracies and anatomical variations occurring during treatment. For instance, anatomical differences in the oral cavity were observed in patient 2HNA011 (Figure 3); these misalignments were propagated to the generated sCT, affecting its apparent visual quality and negatively influencing the quantitative metrics reported in Table 2.

Across the federated centers, performance differences were primarily attributed to image misalignments, despite the inclusion of an additional deformable registration step for the evaluation data. The lower performance at Center B relative to Centers C, E, and the external Center A was mainly attributable to a higher prevalence of misalignments. In contrast, the superior performance at Center C reflected a reduced influence of registration errors, resulting in higher image similarity metrics. Taken together, these observations suggest that registration quality, rather than the inherent capability of the generative model, represented a primary limiting factor in the evaluation. To provide further analytical insights, an additional investigation was conducted on the Center A test cohort using NMI as an intensity-based surrogate for registration quality (see Section 2.5). Although NMI lacks direct geometric quantification of spatial misalignment, it measures intensity correspondence between CT and CBCT volumes. A significant negative correlation was identified between ΔNMI and ΔMAE. Furthermore, Figure 7 supports the hypothesis that residual misalignment systematically affects sCT evaluation. In detail, when deformable registration substantially improved intensity correspondence (ΔNMI≈10%−15%), a corresponding MAE reduction was observed. In contrast, when NMI deteriorated, MAE degraded by approximately 20–60 HU (upper-left area of Figure 7).

However, the robustness of the federated model was further reflected by the results obtained at Center E, where data were acquired using a different scanner and acquisition protocol. Despite this domain shift, the model maintained performance levels consistent with the federation, highlighting its capacity to adapt across heterogeneous scanners. A global analysis across all centers, focusing on anatomical structures and HU distributions, demonstrated that the model preserved tissue-specific intensity patterns (Figures 3–6). The largest errors were consistently observed in tissues occupying smaller volumes in the head and neck region, such as air cavities and dense bone, which were underrepresented in the training data relative to soft tissues. These regions are intrinsically more challenging to predict accurately and, due to their extreme HU values, disproportionately affected image similarity metrics.

A limitation of this study concerns the quantitative evaluation strategy. As demonstrated by the NMI-MAE correlation analysis, residual misalignment systematically affected the MAE. However, its independent contribution could not be isolated precisely. Furthermore, the local test sets comprised four patients per client, with the purpose of assessing the federated model fitting at the client level. This methodological approach inherently introduces higher variability in performance estimates. Nevertheless, the findings in Table 2 and Figures 3–6 demonstrated consistent performance of the federated model across both local and external testing, indicating its capacity to maintain comparable outcomes for the clients and the independent center.

In addition, although the integration of FL-generated sCTs into RT treatment planning represents a potential clinical application of this work, its direct evaluation was not feasible in this study due to the absence of dosimetric and planning data in the SynthRAD2025 dataset, which contains imaging data only. This scenario also reflects a realistic clinical setting in which sensitive RT data are not shared, further underlining the relevance of FL as a framework for privacy-preserving multi-institutional model development. Thus, future research may focus on evaluating the clinical applicability of sCTs generated through FL. Furthermore, FL does not inherently guarantee complete privacy protection. Potential information leakage from shared model updates has been reported in the literature, including through gradient and model inversion attacks (34, 35). Mitigation strategies such as secure aggregation or differential privacy can further reduce these risks (34, 35). While these strategies have not been explicitly examined in this work, they remain an important area for future research in federated sCT generation.

From a methodological perspective, federated and centralized DL represent distinct optimization paradigms rather than interchangeable training strategies. As previously observed in the FedSynthCT-Brain (14) study, methodological configurations that are effective in centralized settings may not necessarily translate directly to federated environments, where statistical heterogeneity, client-specific data distributions, and aggregation constraints fundamentally shape the learning dynamics. Centralized training optimizes performance under full data availability and implicitly benefits from pooled distributions, whereas FL aims to achieve stable cross-institutional performance while preserving data privacy and respecting structural data-sharing constraints. FL also introduces additional communication and synchronization overhead compared to centralized training, which may increase overall training time, although this cost is often offset by the practical benefits of decentralized data access and improved feasibility in multi-institutional settings. Within this framework, moderate center-specific performance trade-offs should be interpreted in light of the federated objective: promoting generalization across heterogeneous institutions rather than maximizing performance on any single site. Federated models may also serve as robust shared initializations that can be further adapted through optional local fine-tuning when institution-specific optimization is required. Accordingly, FL should be regarded not as a substitute for centralized training, but as a complementary collaborative framework tailored to clinical scenarios in which data centralization is impractical or restricted.

Our findings suggest potential directions for future research. Within the GAN paradigm, more targeted training strategies may improve the synthesis of anatomically underrepresented structures. The multi-discriminator framework proposed by Kumar et al. (36) could represent a viable approach. In parallel, task-specific loss formulations, such as those introduced by Liang et al. (37) to improve HU fidelity, noise suppression, and dosimetric accuracy, could be integrated into the FL aggregation scheme. However, this would introduce additional challenges related to client-level heterogeneity and personalization. Furthermore, beyond GAN-based architectures, the investigation of diffusion-based models and hybrid CNN-transformer approaches (38) within a federated framework represents a promising direction for improving both image quality and structural consistency in FL-generated sCTs.

In conclusion, this study demonstrated the feasibility and effectiveness of a cross-silo FL approach for CBCT-to-sCT synthesis in the head and neck region, using the publicly available SynthRAD2025 challenge dataset (26). The federated model generalized effectively across the participating centers and on an external validation cohort, maintaining consistent performance despite differences in imaging protocols, scanner types, and acquisition parameters. This mitigates the impact of institutional heterogeneity, a common limitation in single-center model deployments. Furthermore, these results align with requirements outlined in recent related work (8), which emphasized that a model should ideally be applicable across different centers without the need for conditional tuning.

## Data Availability

Publicly available datasets were analyzed in this study. This data can be found here: https://zenodo.org/doi/10.5281/zenodo.15373853.

## Footnotes

^1^https://gdpr-info.eu/.

^2^https://www.hhs.gov/hipaa/index.html.

^3^https://github.com/phillipi/pix2pix.
