# Supplementary material for: A privacy-preserving federated learning framework for generalizable CBCT to synthetic CT translation in head and neck
Source: Front Digit Health. 2026 Jun 15;8:1812254. doi: 10.3389/fdgth.2026.1812254 (PMC13310887; doi:10.3389/fdgth.2026.1812254)
Supplement: Supplementary file 1 [file Datasheet1.pdf]

A Privacy-Preserving Federated Learning Framework for Generalizable CBCT to Synthetic CT Translation in Head and Neck - Supplementary Material

1 LOCAL EPOCHS INVESTIGATION

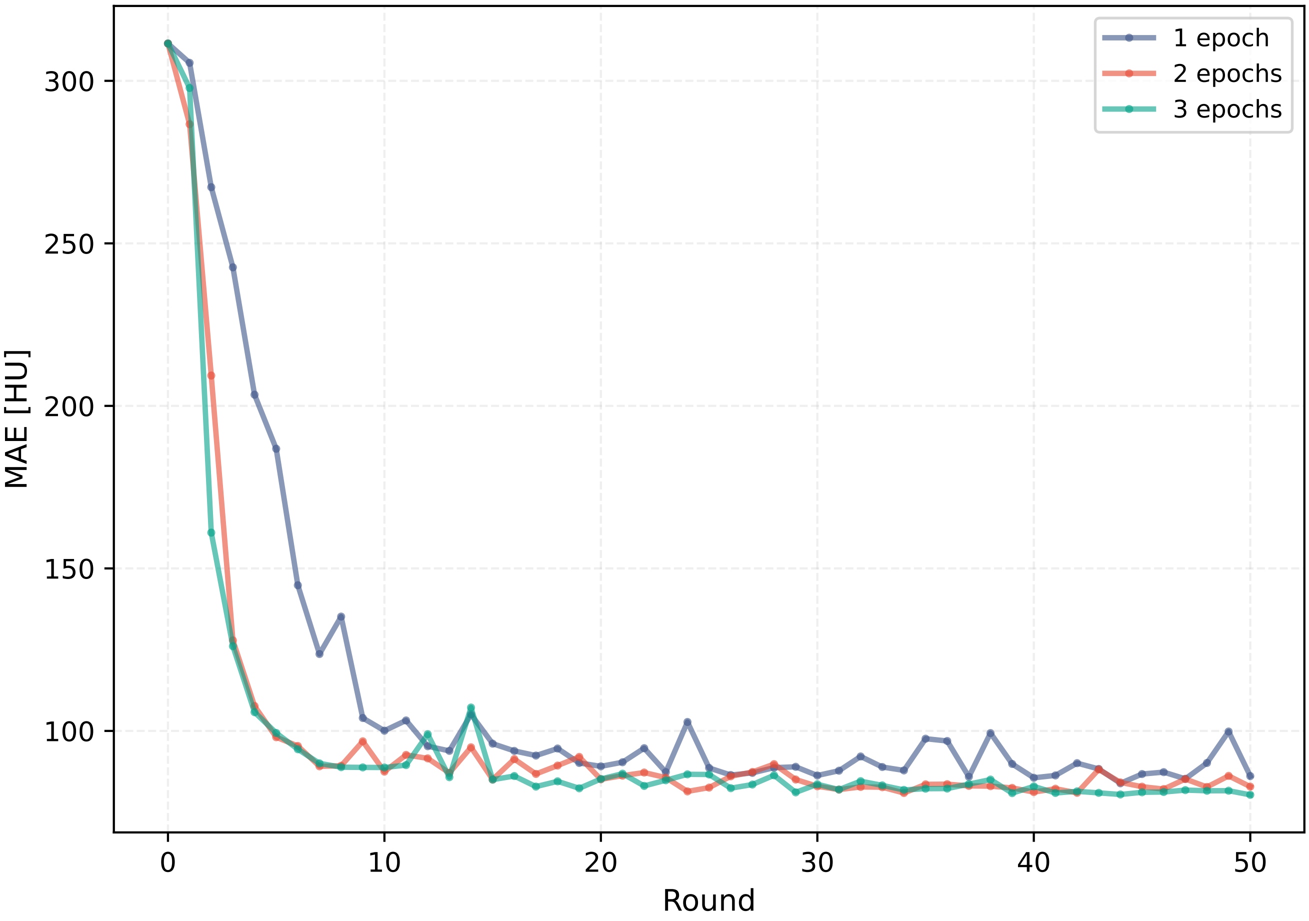

Figure S1. Convergence trend of the global model across federated rounds for different local training configurations (1, 2, and 3 local epochs per round). The MAE decreased substantially in the initial 10 rounds and stabilized at approximately round 30 for configurations with 2 and 3 local epochs. These configurations demonstrated faster and more stable convergence compared to the configuration with a single epoch.

Center	Local Epochs	MAE [HU]	SSIM	PSNR [dB]
Center A (external)	1	77.63 ± 13.29	0.90 ± 0.04	33.58 ± 2.28
	2	75.22 ± 11.81	0.90 ± 0.03	33.52 ± 2.06
	3	74.22 ± 12.34	0.90 ± 0.04	33.74 ± 2.20
Center B	1	83.07 ± 7.65	0.89 ± 0.02	33.24 ± 0.67
	2	85.90 ± 7.10	0.88 ± 0.02	32.86 ± 0.94
	3	81.43 ± 6.44	0.89 ± 0.02	33.15 ± 1.07
Center C	1	65.80 ± 16.17	0.92 ± 0.04	34.42 ± 1.69
	2	64.38 ± 13.63	0.92 ± 0.04	34.48 ± 1.39
	3	64.37 ± 13.91	0.92 ± 0.04	34.47 ± 1.46
Center E	1	73.80 ± 14.66	0.92 ± 0.02	34.89 ± 1.17
	2	74.22 ± 13.23	0.92 ± 0.01	34.91 ± 1.04
	3	76.15 ± 13.86	0.91 ± 0.02	34.69 ± 1.04

Table S1. Quantitative performance across all centers for different local epochs configurations. Results are reported as mean ± standard deviation. Center A was used exclusively for external testing, while Centers B, C, and E participated in the federated training.
